# Inhibition of Carrageenan/Kaolin-Induced Arthritis in Rats and of Inflammatory Cytokine Expressions in Human IL-1β-Stimulated Fibroblast-like Synoviocytes by a Benzylideneacetophenone Derivative

**DOI:** 10.1007/s10753-018-0947-8

**Published:** 2018-12-19

**Authors:** Bongjun Sur, Seungmin Kang, Mijin Kim, Seikwan Oh

**Affiliations:** Department of Molecular Medicine and TIDRC, School of Medicine, Ewha Womans University, Seoul, Republic of Korea

**Keywords:** Arthritis, Benzylideneacetophenone derivative, Anti-inflammatory, Fibroblast-like synoviocytes

## Abstract

The benzylideneacetophenone derivative JC3 [(2E)-3-(4-hydroxy-3-methoxyphenyl)phenylpro-2-en-l-one] (JC3) was synthesized by modifying yakuchinone B obtained from the seeds of *Alpinia oxyphylla*, a member of the ginger family (Zingiberaceae), which are widely used as a folk remedy and as an anti-inflammatory. The aim of this study was to investigate the anti-arthritic effects of JC3 in rat models of carrageenan-induced paw pain and carrageenan/kaolin-induced knee arthritis. The anti-nociceptive effect of JC3 was assessed by measuring paw withdrawal pressure thresholds using an analgesy-meter. Arthritic symptoms in our monoarthritic rat model were evaluated using weight distribution ratios (WDR), paw thicknesses, and serum prostaglandin E2 (PGE2), tumor necrosis factor (TNF)-α, interleukin (IL)-6, and vascular endothelial growth factor (VEGF) levels (determined by ELISA). Histological analyses of knee joints were performed after injecting JC3 intraperitoneally into rats before carrageenan treatment at 5 or 10 mg/kg/day for 6 days. The anti-inflammatory effects of JC3 were investigated *in vitro* using interleukin-1beta (IL-1β)-stimulated fibroblast-like synoviocytes (FLS) derived from arthritis patients. PGE2, IL-6, and IL-8 levels were measured after treating FLS with JC3. In arthritis-induced rats, JC3 treatment significantly decreased nociceptive and arthritic symptoms at days 5 to 6 after carrageenan/kaolin injection. Histological staining of knee tissue showed that JC3 significantly reduced inflammatory areas in the knee joints. Furthermore, JC3 inhibited the expressions of IL-6 and IL-8 in FLS cells at concentrations of 5–10 μg/ml and decreased PGE_2_ levels in FLS cells. These findings suggest JC3 has anti-arthritic effects in *in vivo* and *in vitro*, and that it might be useful for the treatment of arthritis.

## INTRODUCTION

Rheumatoid arthritis (RA) is a response of joint tissues to joint deformation, trauma or biochemical, genetic, or intracellular factors [2, 11]. In arthritis, proliferative fibroblast-like synoviocytes (FLSs) play key roles in joint damage and in the propagation of inflammation because they produce considerable amounts of proinflammatory mediators, such as interleukin-6 (IL-6) and prostaglandin E2 (PGE2) [16]. RA progression is associated with increased cytokine levels of IL-1β, which is produced by macrophages and dendritic cells [20]. Because IL-1β is believed to play a crucial role in synovial inflammation, elevating IL-1β levels in FLSs has been used to mimic the synovial proliferation that occurs in RA [13]. IL-1β is also known to prompt the over expressions of proinflammatory factors in many cell types [3]. IL-1β is an influential inducer of metalloproteinase production by FLSs [6]. The nonsteroidal anti-inflammatory agents used to treat RA are known to have harmful effects, particularly in the gastrointestinal tract, and thus, there is a need for anti-inflammatory drugs with fewer side effects [8, 12].

Yakuchinone B is a constituent of the seeds of *Alpinia oxyphylla* and is a conjugated 1,4-enone containing a phenyl ring, which is a feature of an important class of natural chalcones with wide-ranging biological activities [24]. Furthermore, yakuchinone B is known to have significant biological effects, which include antitumor [5], antiviral [17], and anti-inflammatory [21] effects. The benzylideneacetophenone JC3 was synthesized by modifying yakuchinone B in an effort to develop anti-inflammatory agents [18].

No previous study has been undertaken to investigate the anti-arthritic effects of JC3 on human FLS cells or in an animal model of arthritis. In this study, the anti-arthritic effects of JC3 were evaluated in carrageenan/kaolin-induced rat models and in IL-1β-stimulated rheumatoid arthritis fibroblast-like synoviocytes derived from arthritis patients.

## MATERIALS AND METHODS

### Isolation and Culture of FLSs

From the RA patients who previously had joint replacement surgery, their synovial tissues were used to separate the fibroblast-like synoviocytes (FLSs), which are type of arthritic cells with active inflammation, which then were used to analyze the antiarthritic effect, as described previously [14]. Cells were grown in Dulbecco’s modified Eagle medium (low glucose; Gibco-Invitrogen, Carlsbad, CA, USA) supplemented with 10% (vol/vol) fetal bovine serum (Gibco-Invitrogen), 100 U/mL penicillin, and 100 μg/mL streptomycin sulfate (Gibco-Invitrogen). FLSs of passages 3–6 were used in the experiments.

### Animals

Male Sprague–Dawley rats (Samtaco CO., Osan, Korea) were used throughout. Animals were acclimatized for 1 week before the experiments and housed in an air-conditioned animal room under a 12-h light/dark cycle (08:00–20:00 h light, 20:00–08:00 h dark), at 23 ± 5 °C and 55 ± 10% RH and provided a laboratory diet and water *ad libitum*. The experimental procedures were carried out according to the animal care guidelines of the NIH and the Ewha University Institutional Animal Care and Use Committee.

### Compound Synthesis

JC3 was synthesized as described previously [18]. 4-Hydroxy-3-methoxy cinnamaldehyde was protected with tert-butyldimethylsilyl trifluoromethanesulfonate in the presence of 2,6-lutidine or 2-(trimethylsilyl)ethoxymethyl chloride (SEM–Cl)/*N*,*N*-di-isopropylehylamine to form aldehydes at yields of 95 and 97%, respectively. JC3 was identified by infrared and NMR spectroscopy and high-resolution mass spectroscopy [18].

### Experimental Paw Hyperalgesia and Arthritis Groups

For the paw hyperalgesia model, rats were divided into four groups: the non-treated normal group (the NOR group; *n* = 5), the carrageenan-induced control group (the CON group; *n* = 5), the CON group administered 5 mg/kg of JC3 (the CON+JC3_5 group; *n* = 5), and the CON group administered 10 mg/kg of JC3 (the CON+JC3_10 group; *n* = 5). For the arthritis model, rats were divided into four groups: the non-treated group (the NOR group; *n* = 5), the carrageenan/kaolin-induced control group (the ART group; *n* = 5), the ART group administered 5 mg/kg of JC3 (the ART+JC3_5 group; *n* = 5), and the ART group administered 10 mg/kg of JC3 treatment (the ART+JC3_10 group; *n* = 5).

### Evaluation of Paw Hyperalgesia

To induce paw hyperalgesia, rats were administered an intra-plantar injection of 1% carrageenan (0.1 mL) in the posterior left paw, as described previously [25]. Three hours after injection, pain thresholds were measured using the Randall–Selitto test and a paw pressure analgesia instrument (Ugo Basile Biological Research Apparatus Co., Comeria-Varese, Italy). Ten rats were studied per group and the test was performed blind. Rats were starved overnight and JC3 was evaluated at doses of 5 and 10 mg/kg. JC3 was injected 1 h before carrageenan injection. To evaluate paw hyperalgesia, we measured the tolerance to increasing mild pressure on the affected paw between a flat surface and a blunt pointer of the instrument, according to the manufacturer’s instructions.

### Induction of Arthritis

The 5% carrageenan/kaolin-induced arthritic rat model was prepared as described previously [10]. Animals were briefly anesthetized with 3% isoflurane in a N_2_O/O_2_ mixture. Arthritic inflammation was induced by a single injection of 3% carrageenan/kaolin suspended in 100 μL of pyrogen-free sterile saline, into left knee joints. To evaluate the arthritic progression of carrageenan/kaolin-induced arthritis rat, three different parameters were measured: knee thickness, weight distribution ratio (WDR), and using knee flexion test squeaking scores. These were considered behavioral indicators of carrageenan/kaolin-induced arthritis through checking for 6 days. As arthritis progression, redness and swelling of knee joints and arthritic pain started to appear and reached a maximum on day 1 after the carrageenan/kaolin injection. Daily JC3 injections 5 or 10 mg/kg were started 24 h after the carrageenan/kaolin injection and continued for 5 days. All behavioral tests were performed blinded.

### Evaluation of Knee Thickness

Thicknesses of both hind knees were measured daily using a dial thickness gauge for 6 days after carrageenan/kaolin injection, and mean values were recorded. Thicknesses are expressed as ratios *versus* thicknesses measured prior to carrageenan/kaolin injections.

### WDRs

The WDR is a ratio of the percent of weight carried on each hind leg in which the weight-bearing forces of both hind limbs were measured with an incapacitance meter (Ugo Basile Biological Research Apparatus Co., Comeria-Varese, Italy), as previously described by Hwang et al. [10]. To evaluate arthritic pain, a rat was placed in the test box of an incapacitance meter, which contained a slanted plank is located. The force borne by each hind limb was measured using two mechanotransducers, separately placed below hind limbs. Weights borne by hind limbs were estimated using 5-s averages, and mean weights of four separate estimations were calculated. WDR % was calculated using 100 × (weight borne by ipsilateral limb/total weight borne by both limbs). The WDR of hind paws in the normal group was 50:50, indicating that 50% of the weight was carried in each hind paw. As ankle pain and swelling progressed due to arthritis, this balance moved in favor of contralateral limbs.

### Squeaking Test

Arthritis-induced hyperalgesia was assessed by quantifying the total number of vocalizations evoked by knee flexion or extension. Squeaking scores derived using the modified method of Yu et al. were used to assess knee rigidity and pain while knee joints of hind limbs were gently flexed and extended [26]. The numbers of vocalizations emitted during flexion and extension periods were counted. The cyclic procedure consisting of five 5-s flexion and extension cycles per hind limb. A vocalization rating of 0 (no vocalization) or 1 (vocalization) was given based on response to flexion or extension. Thus, vocalization rating scores lay between 0 and 10 (maximum) for each hind limb. The squeaking test was performed daily for 6 days.

### ELISA

Rat serum samples was obtained by centrifugation at 6500 rpm for 20 min and stored at − 70 °C until use. Levels of the inflammatory mediators IL-6, TNF-α, PGE2, and VEGF were measured using enzyme-linked immunosorbent assay (ELISA) kits (BD Biosciences Pharmingen, San Diego, CA, USA, for IL-6, and TNF-α; R&D Systems, Minneapolis, MN, USA, for PGE2; and Abcam, Cambridge, UK, for VEGF) according to the manufacturer’s instructions as described previously [1]. For the *in vitro* study, triplicate cultures of FLSs were treated with various concentrations of JC3 (1, 5, and 10 μg/mL) and/or IL-1β (10 ng/mL; ProSpec, Rehovot, Israel) and then cultured for 24 h. Supernatants were collected, centrifuged, and analyzed for IL-6, IL-8, and PGE2 expression using commercial ELISA kits, as described previously [1].

### Histological Assessment

For hematoxylin–eosin histochemistry, knee tissues were fixed in 10% paraformaldehyde overnight, dehydrated in 99% ethanol, embedded in paraffin, sectioned at 6 μm (Finesse 325; Thermo Shandon Co., UK), and mounted on slides. Before staining, sections on slides were deparaffinized. To investigate morphologic changes and eosinophil infiltration, sections were stained with hematoxylin (Merck, Darmstadt, Germany) and 1% eosin (Sigma-Aldrich Co., MO, USA), air-dried, and cover-slipped. All slides (× 100 magnification) were observed and photographed using a microscope equipped with camera (BX51; Olympus Ltd., Tokyo, Japan), and images were analyzed using DP2-BSW software (Olympus Ltd., Tokyo, Japan). Subsequently, the stained sections were scored quantitatively in a blinded manner by three independent observers. Histology scores of extent of cellular infiltration and joint integrity were evaluated as follows: 0 = normal; 1 = infiltration of inflammatory cells; 2 = 2 cartilage erosion and pannus formation; 3 = bone erosion; and 4 = bone destruction. The maximum score was 4 per mouse.

### Statistical Analysis

Results are expressed as means ± SEMs. The analysis was conducted by one-way ANOVA using SPSS Ver. 13.0 (SPSS; Chicago, USA). Statistical differences between groups were further analyzed using Tukey’s *post hoc* test. *P* values of < 0.05 were considered statistically significant.

## RESULTS

### Analgesic Effect of JC3 on Carrageenan-Induced Paw Hyperalgesia

**Fig. 1:**
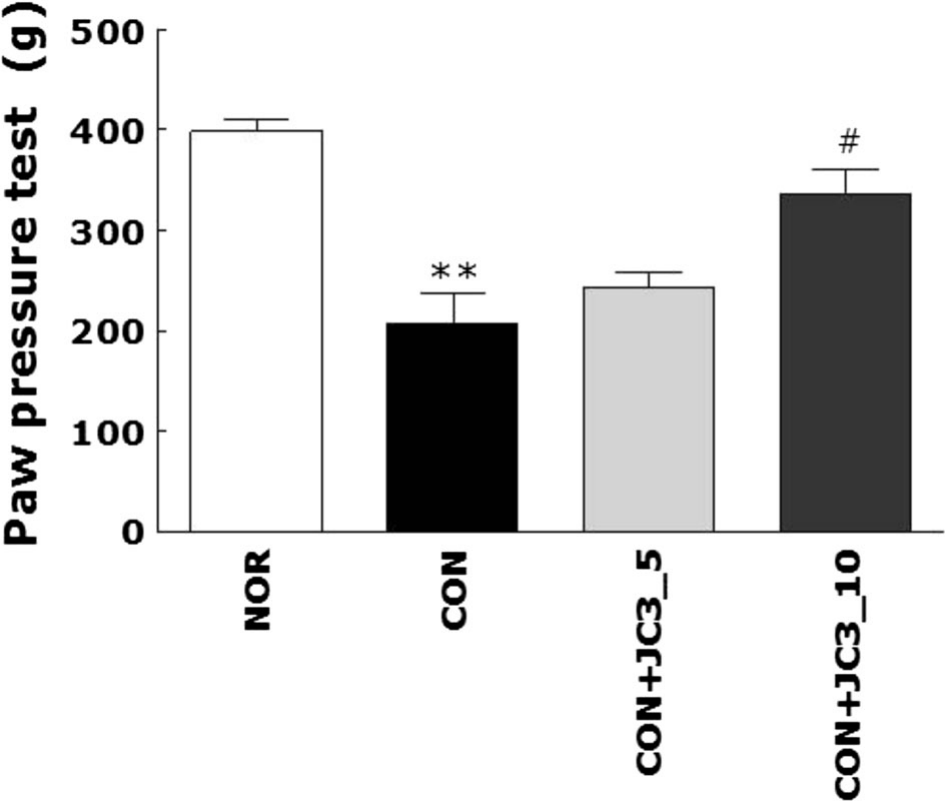
Analgesic effect of JC3 in the carrageenan-induced hyperalgesia rat model. Hyperalgesia was induced by an intra-plantar injection of carrageenan (100 μL of 1% solution) into left hind paws. JC3 was administered (i.p.) 1 h before carrageenan injection. Nociceptive thresholds were determined by the Randall–Selitto test using a paw-pressure analgesy meter 3 h after carrageenan injection. NOR non-treated group, CON carrageenan-treated control group, CON+JC3_5 carrageenan-induced and 5 mg/kg JC3-treated groups, and CON+JC3_10 carrageenan-induced and 10 mg/kg JC3-treated group. Results are presented as means ± standard errors. Data analysis was performed using one-way ANOVA followed by Tukey’s *post hoc* test. **p < 0.01 *vs.* NOR group, #*p* < 0.05 *vs.* CON group.

Treatment with JC3 at 1 h before carrageenan injection increased paw withdrawal thresholds *versus* those observed in the CON group (Fig. 1), indicating JC3 pretreatment inhibited carrageenan-induced hyperalgesia. Furthermore, paw withdrawal thresholds were significantly greater in the CON+JC3_10 group than in the CON group.

### Anti-Arthritic Effect of JC3 on Carrageenan/Kaolin-Induced Arthritis

**Fig. 2:**
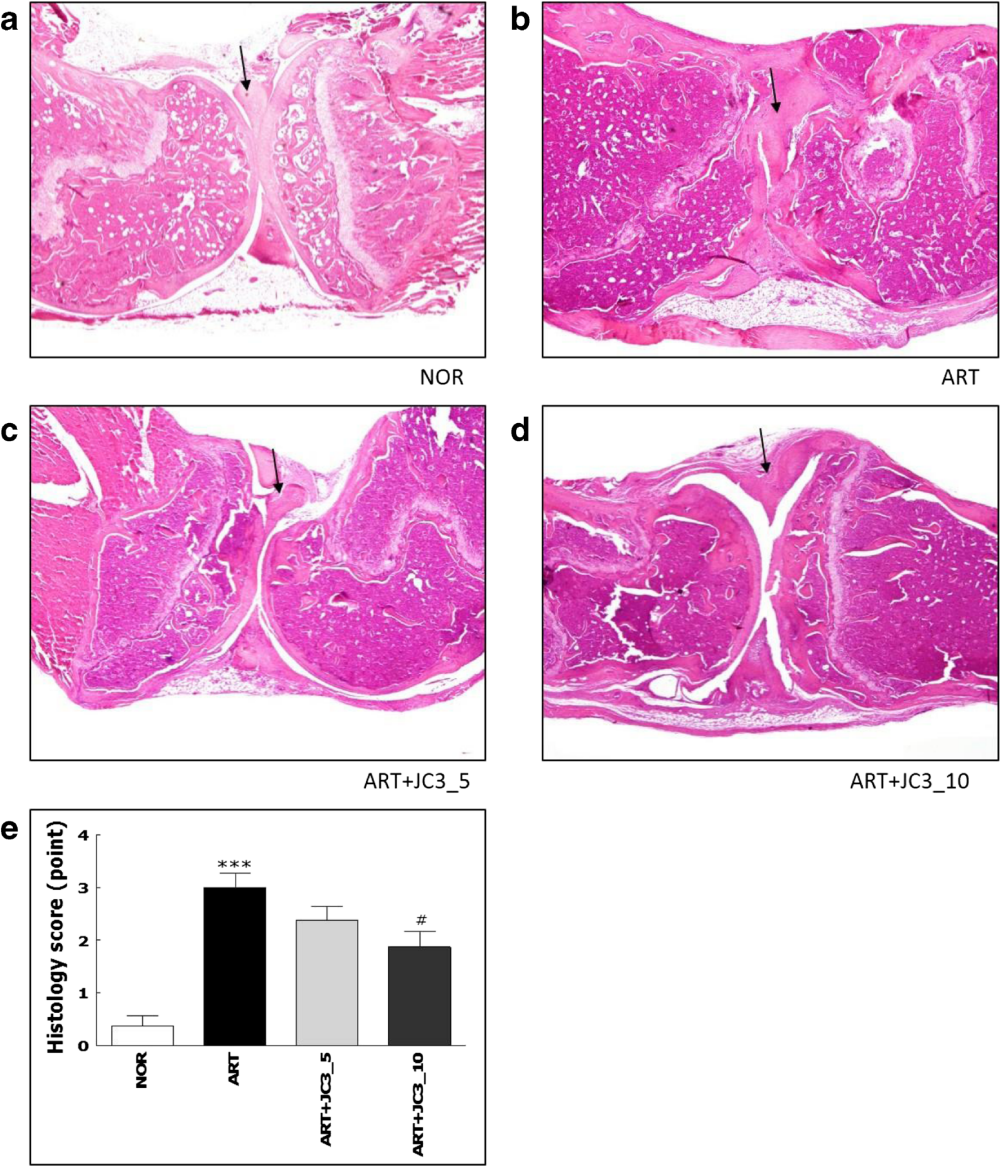
Histological images of the knee joints of rat administered JC3 in the NOR (**a**), ART (**b**), ART+JC3_5 (**c**), and ART+JC3_10 (**d**) groups. **e** Histology scores were determined using a standardized scoring scale as described in “MATERIALS AND METHODS.” Tissues were stained with hematoxylin and eosin (HE). Results are presented as means ± standard errors. ***p < 0.005 *vs.* the NOR group and #*p* < 0.05 *vs.* the ART group.

**Fig. 3:**
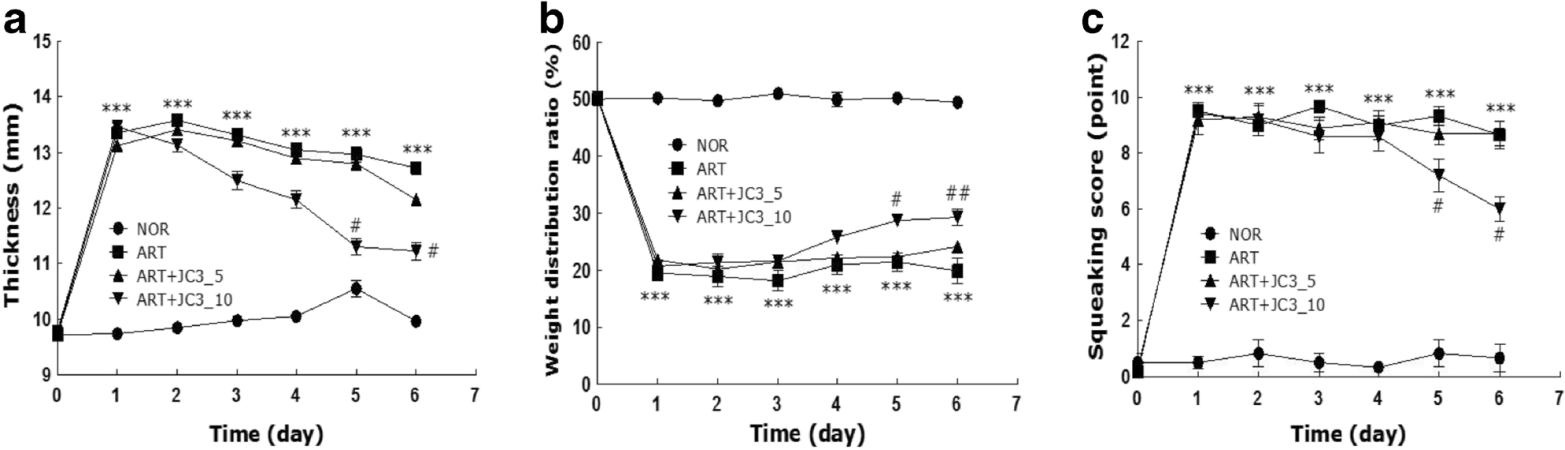
Assessments of anti-arthritic activity in the rat model of carrageenan/kaolin-induced arthritis. **a** Thicknesses of knees, **b** weight distribution ratios (WDRs), and **c** squeaking scores, indicating arthritis severity. Results are presented as means ± standard errors. ***p < 0.005 *vs.* the NOR group and #*p* < 0.05 and ##*p* < 0.01 *vs.* the ART group.

To examine the *in vivo* anti-arthritic effect of JC3, the efficacy of JC3 was evaluated by assessing physical parameters, such as knee morphology, knee thickness, WDR, and squeaking scores. In addition, morphological changes in tissues and inflammatory cell infiltration were also assessed histologically. The ART group exhibited signs of severe inflammation, such as synovial hyperplasia, pannus formation, bone erosion, and cartilage degradation, but these phenomena were inhibited by JC3 treatment (Fig. 2). Knee thickness was used as an indicator of arthritis induced by carrageenan/kaolin treatment, and the ART+JC3_10 group had significantly smaller knee thicknesses than the ART group (Fig. 3a). This result was evidenced by redness and swelling of the entire paw before reaching a plateau, which was steadily maintained throughout the experimental period. Next, we examined the effect of JC3 on WDRs of hind paws of rats with carrageenan/kaolin-induced arthritis (Fig. 3b). At baseline before carrageenan/kaolin injection, mean WDR was 50:50. However, significant changes in WDR were observed on day 1 after carrageenan/kaolin injection, and on day 6, the weight carried by ipsilateral damaged legs in the ART group reached 21%. However, WDR did not deteriorate as much in animals administered 5 or 10 mg/kg JC3, and this effect was significant in the ART+JC3_10.

Vocalization scores for extension and flexion of reached a maximum at day 1 after carrageenan/kaolin injection. In the ART+JC3_10 group, numbers of vocalizations were significantly less at 5 days after injection, whereas little analgesic effect was observed in the ART+JC3_5 group (Fig. 3c).

### Effect of JC3 on Inflammatory Mediators and Angiogenic Mediator in the Serum of Carrageenan/Kaolin-Administered Rats

**Fig. 4:**
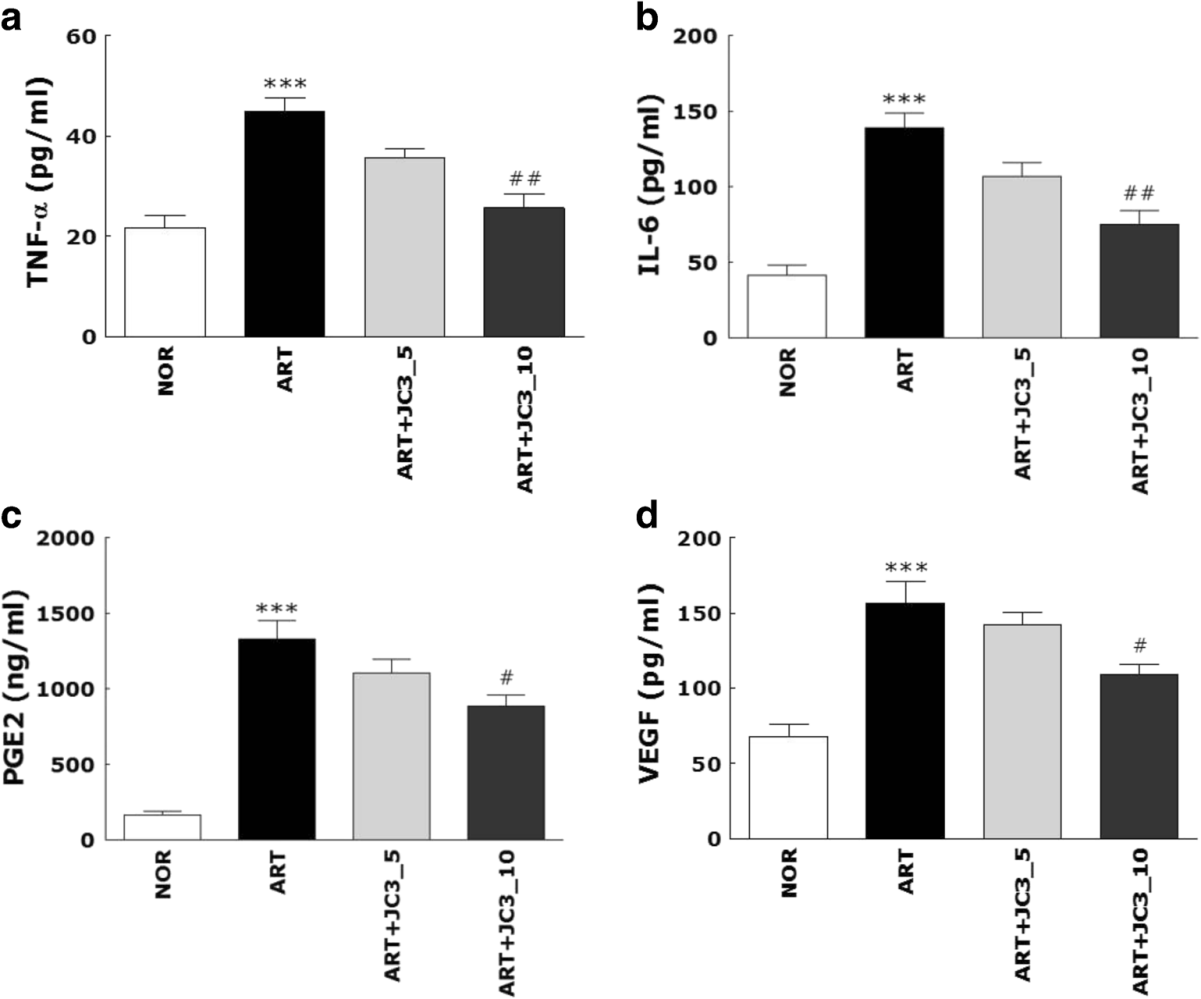
Effects of JC3 on the ELISA determined levels of the pro-inflammatory mediators TNF-α (**a**), IL-6 (**b**), PGE2 (**c**), and VEGF (**d**) in serum of the rat model of carrageenan/kaolin-induced arthritis. Results are presented as means ± standard errors. ***p < 0.005 *vs.* the NOR group and #*p* < 0.05 and ##*p* < 0.01 *vs.* the ART group.

Carrageenan/kaolin injection significantly increased levels of the inflammatory mediators TNF-α, IL-6, and PGE2 and the angiogenic mediator VEGF in serum. An increase in each mediator is an important component of arthritis. Thus, we investigated whether JC3 decreased each mediators in carrageenan/kaolin-induced arthritis rats. A significant increase in serum TNF-α, IL-6, PGE2, and VEGF level was observed in the ART group compared to the NOR group. Expectedly, JC3 was inhibited in TNF-α, IL-6, PGE2, and VEGF level (Fig. 4).

### Effect of JC3 on the Productions of Inflammatory and Angiogenic Mediators in FLSs

**Fig. 5:**
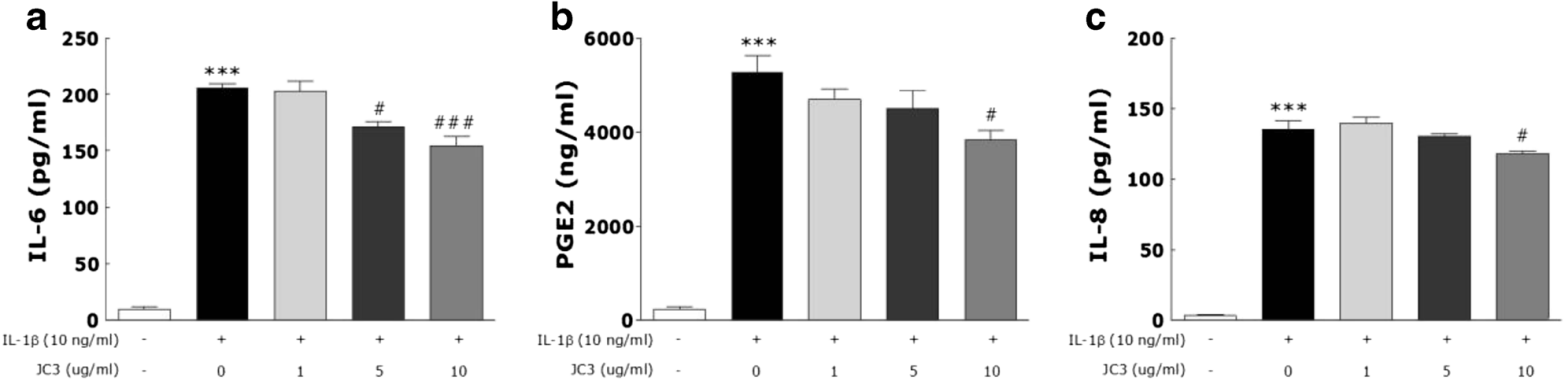
Effects of JC3 on the ELISA determined levels of the pro-inflammatory mediators IL-6 (**a**) and PGE2 (**b**), and on the levels of IL-8 (angiogenic mediator) (**c**) in FLS cells. Results are presented as means ± standard errors. ***p < 0.001 *vs.* nontreated controls, #*p* < 0.05 and ###*p* < 0.005 *vs.* IL-1β-treated cells.

FLSs were treated with IL-1β at 10 ng/mL with or without JC3 (1, 5, and 10 μg/mL). JC3 with or without IL-1β for 24 h did not affect FLS viability as compared with non-treated controls (data not shown). The overexpressions of PGE2, IL-6, and IL-8 in synovium are well known to increase joint destruction and synovial inflammation. Thus, we examined whether JC3 could reduce the IL-1β-induced overexpressions of PGE2, IL-6, and IL-8 in FLSs. Treatment with IL-1β significantly increased PGE2 and IL-6 levels as compared with controls. However, the addition of JC3 inhibited the up-regulations of PGE2 and IL-6 by IL-1β (Fig. 5a, b). In a previous study, angiogenic activities in the conditioned media of inflamed human rheumatoid synovial tissue macrophages and of lipopolysaccharide-stimulated blood monocytes were equally blocked by IL-8 antibody, and an IL-8 antisense oligonucleotide specifically blocked the production of monocyte-induced angiogenic activity. These observations show IL-8 is an important angiogenic factor of the pathogenesis of arthritis. When we examined the effect of JC3, we found it inhibited IL-1β-induced IL-8 secretion in FLSs (Fig. 5c), which suggested JC3 inhibits neovascularization in the inflamed joints of arthritic rats.

## DISCUSSION

Damaging inflammation is the hallmark of RA, and it has become progressively clear even non-inflammatory arthritis is characterized by many progressive immunologically related events. In fact, consequent inflammation in synovial membranes and joint fluid are important symptoms of osteoarthritis, and eventually result in synovitis and synovial hyperplasia. Synovitis is a symptom of inflammatory and degenerative arthritis, and in RA, synovitis and synovial fibroblast activation are key stages in the development of invasive rheumatoid pannus [9].

Synovitis is usually accompanied by proliferation of synovial lining and resultant erosion of bone and joint cartilage. Macrophage-like synoviocytes (MLSs) and FLSs in inflamed synovium play significant roles in the destructive process of joint arthritis. FLSs in inflamed joints secrete inflammatory cytokines, such as IL-6 and IL-8. Furthermore, TNF-α produced by macrophages and FLSs infiltrates synovial linings and contribute to the initiation of joint damage by up-regulating the expressions of various hydrolytic enzymes and pro-inflammatory mediators.

Because of the etiological complexity and diversity of pathological mechanisms in inflammatory arthritis, many researchers have studied non-steroidal anti-inflammatory agents derived from natural sources with the aim of developing new clinical treatments [4]. Yakuchinone B is a constituent of the seeds of *Alpina oxyphylla*, a member of the ginger family (Zingiberaceae), and is commonly used in folk medicine. Yakuchinone B has a variety of biological effects, which include anti-inflammatory activity [22], as demonstrated by its strong inhibitory effect on prostaglandin biosynthesis *in vitro* [15]. In a previous *in vitro* study, it was shown JC3, a synthesized benzylideneacetophenone, has free radical scavenging activity and suppresses lipopolysaccharide (LPS)-induced nitric oxide (NO) generation and anti-excitotoxicity in cortical culture neurons [18]. Based on these results, we assessed the anti-arthritic effects of JC3 to determine whether it had therapeutic potential for the treatment of arthritis.

In this study, we used two animal models to evaluate the *in vivo* analgesic and anti-arthritic effect of JC3. First, we found JC3 significantly ameliorated pain the CON+JC3_10 group in our carrageenan-rat model.

Second, JC3 seemed to inhibit the development of arthritic symptoms as based on knee thicknesses, WDRs, squeaking scores, and ELISA determined TNF-α, IL-6, and PGE2 levels in serum. In the case of knee thicknesses, which were used to assess degree of edema caused by arthritic inflammation, mean knee thickness in the ART+JC3_10 group was less than in the ART group from day 3, and significantly less on day 6. Furthermore, before arthritis induction, mean WDR were 50:50, but from 1 day after carrageenan/kaolin injection, WDR rapidly changed. In the JC3 treatment groups, during the experimental period (6 days), the ratio was continuously balanced. The efficacy of the ART+JC3_10 group was observed after day 4. For these reasons, the efficacy of the JC3 treatment in terms of WDR recovery was significantly verified in this study. In addition, squeaking scores in ART+JC3_10 group were lower than in ART group from day 4, and significantly lower on day 5 and 6.

Next, we found JC3 significantly inhibited the productions of three important proinflammatory mediators, TNF-α, IL-6, and PGE_2_, and inhibited the angiogenic mediator, IL-8, in carrageenan/kaolin-induced rats and IL-1β-stimulated human FLSs. This inhibition of PGE_2_ production is important because PGE_2_ is a known key mediator of inflammatory pain [7]. We also investigated the effect of JC3 on IL-8 (a mediator of angiogenesis) [20] and found that its expression was in the inflammed joints of arthritic rats was inhibited by JC3 treatment.

Finally, the pathological angiogenesis that leads to worsening of arthritis is the critical process [19]. The development of such morbid blood vessel increases the amount of nutrients going into the synovial tissue, increases the movement of immune, and inflammatory cells from the joint, where the inflammation occurred. Due to this result, it becomes the strong provider of the inflammatory cytokine, chemokine, and the growth factor, which then leads to worsening of arthritis [23]. Therefore, when the angiogenesis is inhibited, it inhibits the abnormal growth of inflammation and synovial tissue, and leads to the treatment of arthritis. This study has confirmed decrease in secretion of VEGF of an arthritic animal model with JC3 treatment. Therefore, it is safe to think that this result is shown to have an effect in the treatment of arthritis with JC3.

We reported that JC3 has anti-arthritic effects in animal models and anti-inflammatory effects in IL-1β-stimulated FLSs. As regards the arthritic model used, we found the carrageenan/kaolin injection into the knee joint produced arthritic symptoms, such as edema, pain, stiffness, and rigidity. Collectively, our results suggest that JC3 could be considered as a potential therapeutic agent for the treatment of arthritis.
